# Comparison of Clinical Outcomes Between Left Bundle Branch Area Pacing With a Stylet‐Driven Lead and Conventional Right Ventricular Pacing

**DOI:** 10.1111/jce.16648

**Published:** 2025-03-27

**Authors:** Kyung‐Yeon Lee, Jinsun Park, JungMin Choi, Hyo‐Jeong Ahn, Soonil Kwon, Myung‐Jin Cha, Jun Kim, Gi‐Byoung Nam, Kee‐Joon Choi, Eue‐Keun Choi, Seil Oh, Min Soo Cho, So‑Ryoung Lee

**Affiliations:** ^1^ Department of Internal Medicine Seoul National University Hospital Seoul Republic of Korea; ^2^ Division of Cardiology, Asan Medical Center University of Ulsan College of Medicine Seoul Republic of Korea; ^3^ Division of Cardiology, Department of Internal Medicine SMG–SNU Boramae Medical Center Seoul Republic of Korea; ^4^ Department of Internal Medicine Seoul National University College of Medicine Seoul Republic of Korea

**Keywords:** conduction system pacing left bundle branch area pacing, right ventricular pacing, stylet‐driven lead

## Abstract

**Backgrounds and Aims:**

Left bundle branch area pacing (LBBAP) has been shown to reduce the risk of pacing‐facilitated heart failure (HF) compared to right ventricular pacing (RVP), but limited data exists comparing LBBAP with stylet‐driven leads (SDL) and conventional RVP. The study aims to compare clinical outcomes between LBBAP using SDL and conventional RVP.

**Methods:**

From December 2018 to December 2023, patients who underwent pacemaker implantation at two tertiary hospitals were enrolled. Exclusions included those requiring cardiac resynchronization therapy and patients with ventricular pacing burden ≤ 10%. LBBAP was performed using SDL (Solia S60, Biotronik) with a fixed curve delivery sheath. Composite outcome I consisted of HF admission, pacing‐induced cardiomyopathy (defined as an LVEF decline of ≥ 10% or below 50%), and upgrade to biventricular pacing. Composite outcome II included all‐cause death in addition to the components of composite outcome I.

**Results:**

A total of 738 patients (mean age 72.1 years; 52% of men; 243 LBBAP vs. 495 RVP) were included. Atrioventricular block was more common pacing indication in LBBAP group than RVP group (88.1% vs. 69.3%, *p* < 0.001). Compared to RVP group, ventricular pacing burden was higher in the LBBAP group (96% vs. 86%, *p* < 0.001). LBBAP was associated with a lower risk of composite outcome I and II compared to RVP (adjusted HR 0.27 [95% confidence interval 0.11–0.68], *p* = 0.006 for composite outcome I, aHR 0.41 [0.20–0.84], *p* = 0.015 for composite outcome II), mainly driven by a lower risk of pacing‐induced cardiomyopathy by 70%. There were no significant differences in procedure‐related complications.

**Conclusion:**

LBBAP with SDL was associated with a lower risk of adverse clinical outcomes compared to conventional RVP in patients requiring substantial ventricular pacing.

## Graphical abstract

In this cohort study including 738 patients (243 left bundle branch area pacing using stylet‐driven lead vs. 495 right ventricular pacing), left bundle branch area pacing was associated with a lower risk of the primary outcome by 73% compared to right ventricular pacing with comparable safety profiles.

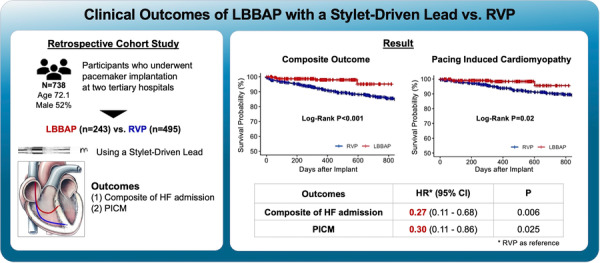

## 1 Introduction

For the treatment of bradyarrhythmia requiring pacemaker, ventricular pacing leads are typically implanted in the right ventricular endocardium; but it is already well‐established that right ventricular pacing (RVP) can cause left ventricular dyssynchrony [1]. This differs from physiological intracardiac conduction and increases the likelihood of pacing‐induced cardiomyopathy (PICM) [2]. Recently, there has been increasing attention towards conduction system pacing (CSP), including His‐bundle pacing (HBP) and left bundle branch area pacing (LBBAP), to preserve the patient's physiologic ventricular activation [3]. Previous observational studies have shown that in patients with a high burden of ventricular pacing (≥ 20–40%), CSP was better for preservation of ventricular contractile function, reducing hospitalization for heart failure (HF), and mortality compared to RVP [4, 5, 6]. Therefore, it has recently been proposed as a primary pacing strategy for patients expected to have a high ventricular pacing burden, particularly in those with decreased left ventricular ejection fraction (LVEF) [7, 8].

Regarding the technical aspects of CSP, HBP has been actively conducted employing a fixed helix lumenless lead (LLL) since the mid‐2010s, while LBBAP has been implemented using the same device from 2017. South Korea presents a unique situation, where adoption of CSP occurred relatively later and only stylet‐driven leads (SDLs) available, prompting physicians to commence CSP via LBBAP utilizing SDL [9], which was different from majority of other countries utilizing LLLs [4, 10, 11, 12]. It has been reported that SDL may generally have a higher rate of microdislodgement compared to LLL [13, 14]; however, most comparative data on LBBAP vs. RVP to date involve LBBAP cohorts implanted primarily with LLL. Although previous reports have indicated comparable procedural success rates and post‐procedural lead profiles between LLL and SDL [15, 16], there is still a scarcity of studies comparing the clinical outcomes with LBBAP with SDL to conventional RVP.

Therefore, to address this clinical unmet need, we conducted an observational study to test the hypothesis that LBBAP with SDL would demonstrate better clinical outcomes compared to RVP.

## 2 Methods

### 2.1 Study Population

**Figure 1:**
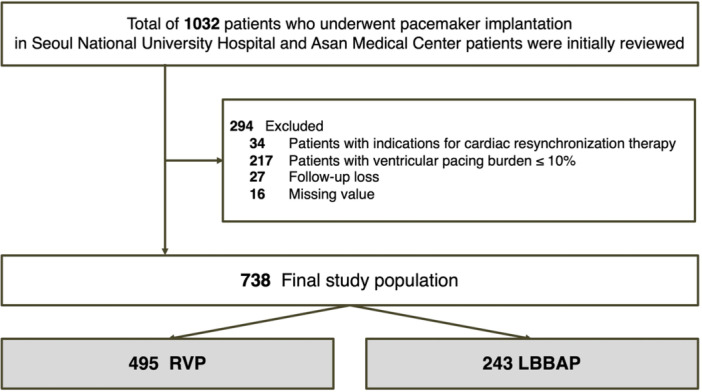
Study flow. LBBAP, left bundle branch area pacing; RVP, right ventricular pacing.

From December 2018 to December 2023, patients who had a pacemaker implanted at Seoul National University Hospital and Asan Medical Center were retrospectively collected. The first case of LBBAP was registered on February 2021, and following the initiation of LBBAP procedures, the majority of pacemaker implantations, especially in patients who were expected high ventricular pacing burden, have shifted from conventional RVP to LBBAP (see Supporting Information S1: Figure S1). To address this issue, the enrollment period for the RVP group precedes that of the LBBAP group (December 2018). A total of 1032 patients were initially included. A total of 294 patients were excluded based on the exclusion criteria, which included patients with indications for cardiac resynchronization therapy (CRT) with biventricular pacing at the time of pacemaker implantation (*n* = 34) and those with ventricular pacing burden ≤ 10% during follow‐up (*n* = 217), along with cases of follow‐up loss (*n* = 27) and missing values (*n* = 16) (Figure 1). This study adhered to the ethical principles outlined in the 2013 revision of the Declaration of Helsinki. The institutional review board at each participating center approved the study, and a waiver of informed consent was granted due to its retrospective design.

### 2.2 Procedure Details of Left Bundle Branch Area Pacing

Single‐ or dual‐chamber pacemakers were implanted according to clinical indications. LBBAP was performed using a SDL with extendable helix (Solia S60, Biotronik) delivered through a fixed curve delivery sheath (Selectra 3D, Biotronik). LBBAP is included left bundle branch pacing (LBBP) and left ventricular septal pacing (LVSP) [8]. The success of LBBAP was defined as meeting at least two of the following criteria [8]: (1) Presence of right bundle branch block (RBBB) configuration observed during unipolar tip pacing. (2) Identification of left bundle branch (LBB) potential with an LBB‐V interval ranging from 15 to 35 ms (in cases with a fascicular potential to QRS interval (1–25 ms) were considered to meet the LBBAP criteria [17]). (3) Transition observed from nonselective LBB capture to selective LBB capture. (4) Shift from nonselective LBB capture to left septal capture at near threshold outputs. (5) Short and consistent R‐wave peak time (RWPT), demonstrated by the stimulus to the peak of the R wave in leads V5 or V6 (RWPT) being less than 75 ms in patients without left bundle branch block (LBBB) and less than 85 ms in patients with LBBB. (6) Utilization of programmed (extra‐stimulus testing) deep septal stimulation to distinguish between LV septal capture and nonselective LBB capture. Patients who underwent deep septal pacing (DSP) were included in the RVP group, based on previous studies that demonstrated the pacing parameters and clinical outcomes of DSP were similar to those of RVP, and there was a significant difference between LBBAP and DSP in the previous reports [18].

### 2.3 Covariates

Clinical details, including age, gender, indication of pacemaker implantation, and comorbidities such as hypertension, diabetes mellitus, chronic kidney disease, HF, structural heart disease, ischemic heart disease, valvular heart disease, atrial fibrillation, as well as physical assessment data and laboratory test results such as QRS duration, underlying bundle branch block, N‐terminal pro b‐type natriuretic peptide (NT‐proBNP) level, serum creatinine level, LVEF, left ventricular end‐diastolic dimension, and left atrium (LA) size, were collected from the medical records at the time the patients were admitted for pacemaker implantation. Presence of HF was identified based on the medical records, structural heart disease was included the presence of congenital heart disease, ischemic and nonischemic cardiomyopathy, valvular heart disease, and individuals who have undergone cardiac surgery. Valvular heart disease was defined as the presence of moderate or greater degree of valvular disease, or undergoing valve surgery. The covariates of current medication included antiarrhythmic drug, beta‐blockers, renin‐angiotensin system blockers, and diuretics.

### 2.4 Clinical Outcomes

The primary outcome was defined as composite outcome I, which included PICM, hospitalization or unplanned hospital visits for HF, and device upgrade to CRT. Additionally, we analyzed composite outcome II, which was defined as composite outcome I with the addition of all‐cause death. The secondary outcomes include each component of the primary outcome as well as cardiovascular death, all‐cause death, and procedure related complications. PICM was defined as a decline in LVEF from normal to EF < 50% or a decrease of more than 10% (as an absolute value) from baseline [19]. HF Hospitalization was defined as an unplanned hospital visit including emergency department visit, or hospitalization due to HF symptoms requiring intravenous diuretics administration [20]. An upgrade to CRT was defined as the replacement of an existing pacemaker with a CRT device when the patient met the established clinical criteria for CRT implantation. Cardiovascular death was defined as death resulting from HF, coronary artery disease, myocardial infarction, cardiogenic shock, or cardiac arrest due to fatal ventricular arrhythmias, including ventricular tachycardia and ventricular fibrillation [21]. Regarding the acute procedural complication, ventricular lead‐related complication was defined as a composite of lead fracture during implantation or severe right ventricular lead‐induced tricuspid regurgitation requiring lead removal. Pocket‐related complication was defined as an infection of the pocket or lead, or the occurrence of a pocket hematoma that required additional intervention. For chronic complications, the need for ventricular lead revision following discharge from the index hospitalization was assessed.

### 2.5 Statistical Analysis

Baseline characteristics between the two groups was compared using Student's *t*‐test for continuous variables, as mean ± standard deviation or median (interquartile range, IQR), and the *χ* ^2^ test for categorical variables. The incidence rate was calculated as the number of events for the first occurrence of each outcome per 100 person‐years at risk. Survival analysis between the two groups was conducted using the Kaplan–Meier method and Cox proportional hazard regression analysis, with the risk of reaching the study endpoints between the two groups presented as hazard ratios (HRs) with 95% confidence intervals (CIs). The multivariable model was used to calculate adjusted HRs and CIs for the covariates. Unadjusted HRs (Model 1); HRs adjusted for age and sex (Model 2). We incorporated clinically relevant variables and those exhibiting a *p*‐value < 0.1 between the two groups in our cohort into the adjusted Model 3. This model accounted for HRs adjusted for age, sex, hypertension, diabetes mellitus, HF, valvular heart disease, atrial fibrillation, diuretics, baseline QRS duration, and underlying BBB. A statistical significance threshold of *p*‐value < 0.05 was applied. All statistical analyses were conducted using R programming version 4.3.2 (The R Foundation for Statistical Computing, Vienna, Austria, http://www.R-project.org).

### 2.6 Sensitivity Analyses

Sensitivity analyses were performed to account for the different follow‐up durations between the two groups, and the adjusted HR (Model 3) was presented by restricting follow‐up to 1 and 2 years, respectively. Additional sensitivity analyses were conducted in subpopulation with atrioventricular block (AVB), dual chamber pacing, and those who did not undergo cardiac surgery.

As a retrospective study, we recruited RVP cohort before (mainly) and after the introduction of LBBAP to ensure a sufficient number of control cases (RVP group). Given the potential selection bias affecting patients who underwent RVP despite the availability of LBBAP, we conducted a sensitivity analysis by restricting the RVP group to the cohort before the introduction of LBBAP.

Furthermore, in this study, patients who successfully underwent LBBAP were classified into the LBBAP group, while those who attempted but failed LBBAP, resulting in DSP, were classified into the RVP group. Since these patients were not initially intended for RVP but rather experienced LBBAP failure, they may have had underlying cardiac structural abnormalities that made LBBAP unfeasible or represented a more ill patient population. Therefore, we planned to conduct a sensitivity analysis excluding these patients from the RVP group and present the results accordingly.

## 3 Results

### 3.1 Baseline Characteristics

A total of 738 study patients (495 patients in RVP group and 243 patients in LBBAP group) were included. The procedural success rate of LBBAP was 91% in our cohort. Baseline characteristics are presented in Table 1. The mean age was 72.1 ± 12.3 years and 51.6% were men. Regarding the indication of permanent pacemaker implantation, LBBAP group was included more patients with atrioventricular block than the RVP group (88.1% vs. 69.3%, *p* < 0.001). Dual chamber pacemaker implantation was more common in the LBBAP group than the RVP group (91.4% vs. 74.1%, *p* < 0.001). There were no significant differences in age, sex, and comorbidities, including hypertension, diabetes, chronic kidney disease, HF, structural heart disease, and ischemic heart disease between the two groups. Repaired valve and AF were more prevalent in the RVP group, and moderate to severe valvular heart disease was more prevalent in LBBAP group. The RVP group showed a higher proportion of use of antiarrhythmic drugs and diuretics. Baseline QRS was wider (121.1 ± 32.4 ms vs. 114.0 ± 28.8 ms, *p* = 0.004), and the proportion of LBBB was higher (6.5% vs. 13.6%, *p* < 0.001) in the LBBAP group. In the echocardiography, LA was smaller in the LBBAP group, whereas LVEF and LVEDD were similar between groups.

**Table 1:** Baseline characteristics for left bundle branch area pacing and right ventricular apex groups.

	Total (*n* = 738)	RVP (*n* = 495)	LBBAP (*n* = 243)	*p*‐value
Age, year	72.1 ± 12.3	72.5 ± 11.6	71.5 ± 13.4	0.312
Sex, male	381 (51.6)	251 (50.7)	130 (53.5)	0.526
Indication				< 0.001
Atrioventricular block	557 (75.5)	343 (69.3)	214 (88.1)	
Sick sinus syndrome	135 (18.3)	118 (23.8)	17 (7.0)	
AF SVR	46 (6.2)	34 (6.9)	12 (4.9)	
Chamber				< 0.001
Single chamber	149 (20.2)	128 (25.9)	21 (8.6)	
Dual chamber	589 (79.8)	367 (74.1)	222 (91.4)	
Comorbidities				
Hypertension	377 (51.1)	259 (52.3)	118 (48.6)	0.377
Diabetes mellitus	183 (24.8)	112 (22.6)	71 (29.2)	0.063
Chronic kidney disease	209 (28.3)	133 (26.9)	76 (31.3)	0.245
Heart failure	64 (8.7)	44 (8.9)	20 (8.2)	0.873
Structural heart disease	152 (20.6)	105 (21.2)	47 (19.3)	0.622
Ischemic heart disease	120 (16.3)	78 (15.8)	42 (17.3)	0.673
Valvular heart disease				0.013
Moderate to severe grade	153 (20.7)	91 (18.4)	62 (25.5)	
Repaired valve	79 (10.7)	62 (12.5)	17 (7.0)	
AF				< 0.001
None	515 (69.8)	320 (64.6)	195 (80.2)	
Paroxysmal AF	123 (16.7)	99 (20.0)	24 (9.9)	
Persistent AF	100 (13.6)	76 (15.4)	24 (9.9)	
Medication				
Antiarrhythmic drug	43 (5.8)	38 (7.7)	5 (2.1)	0.004
Beta‐blocker	107 (14.5)	66 (13.3)	41 (16.9)	0.241
RAS blocker	212 (28.7)	151 (30.5)	61 (25.1)	0.151
Diuretics	153 (20.7)	116 (23.4)	37 (15.2)	0.013
Baseline QRS duration, ms	116.4 ± 30.2	114.0 ± 28.8	121.1 ± 32.4	0.004
Underlying bundle branch block				< 0.001
None	432 (58.5)	304 (61.4)	128 (52.7)	
Left bundle branch block	65 (8.8)	32 (6.5)	33 (13.6)	
Right bundle branch block	192 (26.0)	136 (27.5)	56 (23.0)	
NSIVCD	17 (2.3)	9 (1.8)	8 (3.3)	
Paced beat (temporary pacing)	32 (4.3)	14 (2.8)	18 (7.4)	
Laboratory findings				
NT‐proBNP (pg/mL)	2325.8 ± 7079.3	2311.3 ± 7745.2	2356.7 ± 5428.8	0.955
Creatinine (mg/dL)	0.9 ± 0.9	0.9 ± 1.0	0.9 ± 0.8	0.687
Echocardiography parameter				
LVEF, %	61.2 ± 7.0	61.2 ± 7.0	61.2 ± 7.0	0.995
LVEDD, mm	49.7 ± 6.1	49.8 ± 5.9	49.4 ± 6.4	0.377
LA size, mm	44.9 ± 8.8	45.8 ± 9.4	43.0 ± 7.1	< 0.001

Abbreviations: AF, atrial fibrillation; ECG, electrocardiogram; IQR, interquartile; LA, left atrium; LBBAP, left bundle branch area pacing; LVEDD, left ventricular end diastolic diameter; LVEF, left ventricular ejection fraction; NSIVCD, nonspecific intraventricular conduction delay; NT‐proBNP, N‐terminal pro B‐type natriuretic peptide; RAS, renin‐angiotensin system; RVP, right ventricular pacing; SVR, slow ventricular response.

### 3.2 Procedure Characteristics and Ventricular Pacing Parameters

**Figure 2:**
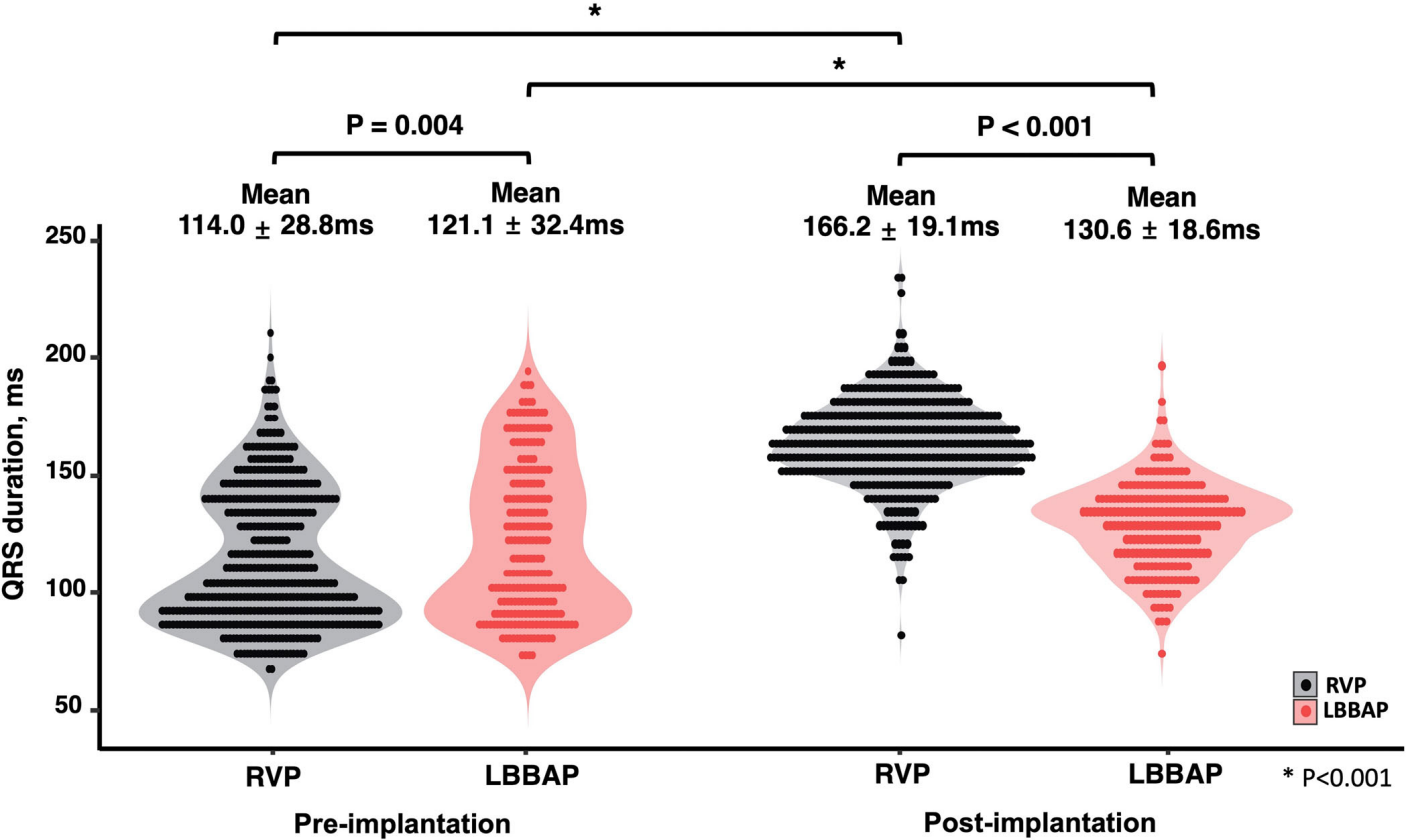
The changes of paced QRS duration according to the pacing site. LBBAP, left bundle branch area pacing; RVP, right ventricular pacing.

Procedure characteristics and ventricular pacing parameters by pacing site at the time of implantation are shown in Table 2. LBBAP group showed slightly longer mean procedure and fluoroscopic time compared to the RVP group. The mean paced QRS duration was significantly narrower in the LBBAP group than in the RVP group (130.6 ± 18.6 ms vs. 166.2 ± 19.1 ms, *p* < 0.001, Table 2 and Figure 2). At immediate after procedure, ventricular pacing thresholds and R‐wave amplitudes were slightly higher in the LBBAP group than in the RVP group. The mean burden of ventricular pacing which was based on the results of interrogation from the 3‐month to 1‐year follow‐up period was significantly higher in the LBBAP group compared to the RVP group (95.5 ± 13.5% vs. 86.3 ± 25.4%, *p* < 0.001). At 12‐month follow‐up, ventricular pacing thresholds decreased in the LBBAP group (median change [IQR], −0.2 [−0.4; 0.1] V), increased in the RVP group (median change [IQR], 0.1 [0.0; 0.2] V) over time, and the mean ventricular pacing threshold at 12‐month was slightly higher in LBBAP group than RVP group (0.9 ± 0.2 V vs. 0.8 ± 0.3 V, *p* < 0.001, Table 3). The R‐wave amplitude was higher at baseline in the LBBAP group, but there was no significant difference at 12‐month (*p* = 0.083), and lead impedance showed no significant difference in pacing thresholds at implantation and at 12‐month (Table 3).

**Table 2:** Procedure data and baseline pacing parameters at implant.

	Total (*n* = 738)	RVP (*n* = 495)	LBBAP (*n* = 243)	*p*‐value
Procedure time, min	53.9 ± 26.7	50.9 ± 25.5	60.0 ± 27.9	< 0.001
Fluoroscopic time, min	9.7 ± 8.7	9.2 ± 9.4	10.7 ± 7.2	0.022
Paced QRS duration, ms	154.2 ± 25.3	166.2 ± 19.1	130.6 ± 18.6	< 0.001
Threshold at implant, V	0.7 ± 0.5	0.7 ± 0.5	0.9 ± 0.3	< 0.001
R‐wave at implant, mV	10.0 ± 4.7	9.5 ± 4.8	11.2 ± 4.1	< 0.001
Impedance at implant, ohm	693.8 ± 237.2	699.5 ± 165.6	682.3 ± 339.2	0.455
Pacing burden (%)^a^	89.4 ± 22.6	86.3 ± 25.4	95.5 ± 13.5	< 0.001

Abbreviations: LBBAP, left bundle branch area pacing; RVP, right ventricular pacing.

^a^ The pacing burden is based on the results of interrogation from the 3‐month to 1‐year follow‐up period.

**Table 3:** Pacing parameter changes from baseline to 12‐month.

	RVP (*n* = 495)			LBBAP (*n* = 243)			*p*‐value^a^
	Baseline	12‐month	Change	Baseline	12‐month	Change	
*Threshold, V*							
Mean ± SD	0.7 ± 0.5	0.8 ± 0.3	0.1 ± 0.4	0.9 ± 0.3	0.9 ± 0.2	−0.2 ± 0.4	< 0.001
Median [IQR]	0.5 [0.5; 0.8]	0.8 [0.6; 0.9]	0.1 [0.0; 0.2]	0.8 [0.6; 1.1]	0.9 [0.7; 1.0]	−0.2 [−0.4; 0.1]	
*R‐wave, mV*							
Mean ± SD	9.5 ± 4.8	11.0 ± 4.8	2.0 ± 4.7	11.2 ± 4.1	13.6 ± 4.8	3.0 ± 4.6	0.083
Median [IQR]	8.9 [6.0; 12.0]	11.4 [7.8; 12.6]	1.5 [−0.2; 4.0]	10.5 [8.1; 13.2]	12.9 [10.3; 16.8]	3.1 [−0.2; 5.2]	
*Impedance, ohm*							
Mean ± SD	699.5 ± 165.6	582.4 ± 146.7	−140.7 ± 184.9	682.3 ± 339.2	578.3 ± 145.1	−128.6 ± 163.8	0.460
Median [IQR]	684.0 [580.0; 810.0]	565.0 [480.0; 646.0]	−117.0 [−212.0; −38.0]	643.0 [565.0; 770.0]	565.0 [526.0; 604.0]	−163.0 [−225.0; −77.0]	

Abbreviations: IQR, interquartile range; LBBAP, left bundle branch area pacing; RVP, right ventricular pacing; SD, standard deviation.

^a^ *p*‐value was calculated based on the mean ± SD of the 12‐month values for each variable between the two groups.

### 3.3 Clinical Outcomes

The median follow‐up duration was 568.0 days (IQR 295.0–911.0 for total study population; 776.0 days [IQR, 381.0–1032.5] for RVP group and 371.0 days [IQR 213.5–557.5] for LBBAP group [*p* < 0.001]). The crude event numbers of clinical outcomes during follow‐up were presented in Supporting Information S1: Table S1.

**Figure 3:**
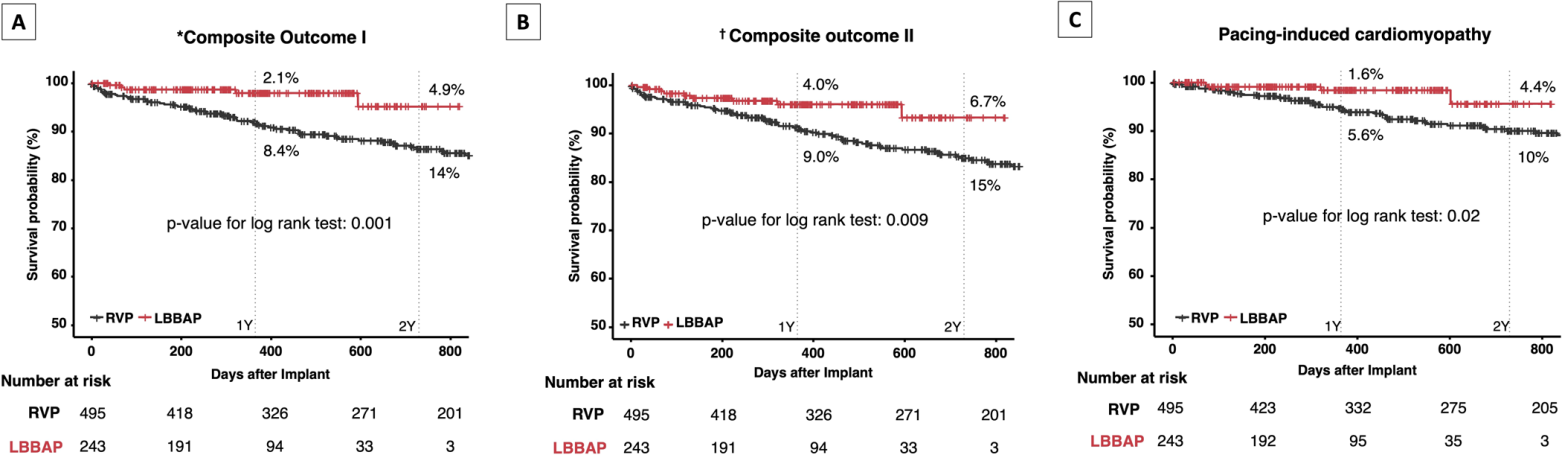
The Kaplan–Meier curves of composite outcome and pacing‐induced cardiomyopathy according to the pacing site. (A) LBBAP showed a significantly lower risk of Composite Outcome I compared to RVP. The 1‐year event rates were 2.1% and 8.4%, respectively, while the 2‐year event rates were 4.9% and 14% (*p*‐value for log‐rank test: 0.001). (B) LBBAP showed a significantly lower risk of Composite Outcome II compared to RVP. The 1‐year event rates were 4.0% and 9.0%, respectively, while the 2‐year event rates were 6.7% and 15% (*p*‐value for log‐rank test: 0.009). (C) LBBAP showed a significantly lower risk of pacing‐induced cardiomyopathy compared to RVP. The 1‐year event rates were 1.6% and 5.6%, respectively, while the 2‐year event rates were 4.4% and 10% (*p*‐value for log‐rank test: 0.02). *Composite outcome I included PICM, hospitalization or unplanned hospital visits for HF, and device upgrade to CRT. ^†^Composite outcome II included PICM, hospitalization or unplanned hospital visits for HF, device upgrade to CRT and all‐cause death. LBBAP, left bundle branch area pacing; RVP, right ventricular pacing.

For the composite outcome I, which included PICM, unplanned hospital visits or hospitalization for HF, and upgrade to CRT, LBBAP group showed significantly lower incidence rate than RVP group (2.06 vs. 6.83 per 100 person‐years, *p* < 0.001, Table 4). The LBBAP group was associated with a significantly lower incidence rate of the composite outcome II (which includes all‐cause death in addition to composite outcome I) compared to the RVP group (3.70 vs. 7.82 per 100 person‐years, *p* = 0.015) (Table 4). For the secondary outcomes, LBBAP group generally showed numerically lower incidence rates than RVP group across all outcomes (Table 4). Among these outcomes, LBBAP group demonstrated a statistically significant lower incidence rate of PICM compared to the RVP group (1.63 vs. 4.48 per 100 person‐years, *p* < 0.001, Table 4). The Kaplan–Meier curves for composite outcome I, composite outcome II, and PICM, presented in Figure 3, indicate that LBBAP was significantly associated with a lower risk of composite outcome I (log‐rank *p* = 0.001), composite outcome II (log‐rank *p* = 0.009), and PICM (log‐rank *p* = 0.02) during follow‐up. After multivariable adjustment (Model 3), the LBBAP group had a 73% lower risk of composite outcome I (adjusted HR [aHR] 0.27, 95% CI 0.11–0.68, *p* = 0.006) and a 59% lower risk of composite outcome II (aHR 0.41, 95% CI 0.20–0.84, *p* = 0.015) compared to the RVP group (Table 4 ). Among the secondary outcomes, LBBAP group was associated with a lower risk of PICM by 70% than RVP group (adjusted HR 0.30, 95% CI 0.11–0.86, *p* = 0.025, Table 4).

**Table 4:** Crude incidences and hazard ratios for clinical outcomes according to the pacing site.

	Event/*N*	IR (100PY)	Model 1		Model 2		Model 3	
			HR (95% CI)	*p*‐value	HR (95% CI)	*p*‐value	HR (95% CI)	*p*‐value
Composite outcome I^a^								
RVP	63/495	6.83	1 (Reference)	—	1 (Reference)	—	1 (Reference)	—
LBBAP	5/243	2.06	0.25 (0.10–0.63)	0.003	0.25 (0.10–0.62)	0.003	0.27 (0.11–0.68)	0.006
Composite outcome II^b^								
RVP	72/495	7.82	1 (Reference)	—	1 (Reference)	—	1 (Reference)	—
LBBAP	9/243	3.70	0.4 (0.20–0.82)	0.012	0.40 (0.20–0.81)	0.011	0.41 (0.20–0.84)	0.015
HF hospitalization								
RVP	38/495	3.84	1 (Reference)	—	1 (Reference)	—	1 (Reference)	—
LBBAP	5/243	2.01	0.43 (0.17–1.12)	0.084	0.43 (0.17–1.12)	0.085	0.49 (0.19–1.28)	0.145
PICM								
RVP	42/495	4.48	1 (Reference)	—	1 (Reference)	—	1 (Reference)	—
LBBAP	4/243	1.63	0.30 (0.11–0.84)	0.022	0.29 (0.10–0.83)	0.021	0.30 (0.11–0.86)	0.025
CRT upgrade			N/A		N/A		N/A	
RVP	7/495	0.68	—	—	—	—	—	—
LBBAP	0/243	0	—	—	—	—	—	—
CV death			N/A		N/A		N/A	
RVP	7/495	0.68	—	—	—	—	—	—
LBBAP	0/243	0	—	—	—	—	—	—
All‐cause death								
RVP	22/495	2.14	1 (Reference)	—	1 (Reference)	—	1 (Reference)	—
LBBAP	5/243	1.97	0.87 (0.32–2.42)	0.795	0.88 (0.32–2.44)	0.809	0.89 (0.32–2.52)	0.833

*Note:* Model 1: Unadjusted. Model 2: Adjusted by age and sex. Model 3: Adjusted by age, sex, hypertension, diabetes mellitus, heart failure, valvular heart disease, atrial fibrillation, diuretics, baseline QRS duration, and underlying bundle branch block.

Abbreviations: CI, confidence interval; CRT, cardiac resynchronization therapy; CV, cardiovascular; HF, heart failure; HR, hazard ratio; IR, incidence rate; LBBAP, left bundle branch area pacing; N/A, not available; PICM, pacing‐induced cardiomyopathy; PY, person‐year; RVP, right ventricular pacing.

^a^ Composite outcome I included PICM, hospitalization or unplanned hospital visits for HF, and device upgrade to CRT.

^b^ Composite outcome II included PICM, hospitalization or unplanned hospital visits for HF, device upgrade to CRT and all‐cause death.

### 3.4 Sensitivity Analyses

The results of sensitivity analyses were consistent with the main results (Supporting Information S1: Tables S3, S4, S5, and S6). When the follow‐up duration was restricted to 1 year, the risk of primary outcomes was statistically significantly lower in the LBBAP group compared to the RVP group (aHR 0.04, 95% CI 0.00–0.37, *p* = 0.004 for the composite outcome I, and aHR 0.29, 95% CI 0.009–0.96, *p* = 0.043 for the composite outcome II), and the trend of the results was consistent with the main analysis when restricted to 2 years (Supporting Information S1: Table S3).

In subpopulation with AVB, LBBAP was significantly associated with a lower risk of the composite outcome I (aHR 0.21, 95% CI 0.06–0.71, *p* = 0.012), and the composite outcome II (aHR 0.43, 0.19–0.98, *p* = 0.043) than the RVP group (Supporting Information S1: Table S4). In those with dual camber pacing, LBBAP was also significantly associated with a lower risk of composite outcome I (aHR 0.17, 95% CI 0.05–0.55, *p* = 0.003) and composite outcome II (aHR 0.36, 95% CI 0.16–0.83, *p* = 0.016) (Supporting information S1: Table S4). In those without prior cardiac surgery, the results of the main analysis were consistently observed (aHR 0.22, 95% CI 0.08–0.61, *p* = 0.004) for the composite outcome I, and aHR 0.35, 95% CI 0.16–0.79, *p* = 0.011 for the composite outcome II) (Supporting Information S1: Table S4).

Additionally, a sensitivity analysis, excluding patients in the RVP group who underwent RVP after the introduction of LBBAP, showed a trend consistent with the main results (Supporting Information S1: Table S5). The sensitivity analysis excluding patients in the RVP group who attempted but failed LBBAP, resulting in DSP, showed results consistent with the main analysis (Supporting Information S1: Table S6).

### 3.5 Safety Outcomes

Acute complications including ventricular pacing lead‐related complication, pneumothorax, pericardial effusion, and pocket related complication did not differ between the two groups (Supporting Information S1: Table S2). There was no occurrence of ventricular pacing lead related complication in both groups. Pneumothorax occurred in 1 and 2 patients in RVP and LBBAP group, respectively. Pericardial effusion occurred in 4 and 0 patients of RVP and LBBAP group, respectively, with all cases required pericardiocentesis. A pocket‐related complication, specifically implant site hematoma, occurred in 2 patients of the RVP group and was resolved with compression. Ventricular lead re‐intervention was required in 15 patients in the RVP group and 3 patients in the LBBAP group. In the LBBAP group, the reasons for ventricular lead re‐intervention were lead dislodgement in two patients and device infection in one patient.

## 4 Discussion

This observational cohort study, utilizing data from two tertiary centers in South Korea, aimed to compare the procedural and clinical outcomes of LBBAP with SDL and conventional RVP. We found that (1) the LBBAP group was associated with a 73% lower risk of composite outcome I (a composite of PICM, hospitalization or unplanned hospital visits for HF, and device upgrade to CRT) and a 59% lower risk of composite outcome II, which included all‐cause death in addition to composite outcome I, compared to the RVP group, despite having a higher ventricular pacing burden during follow‐up; (2) the results was driven by a lower risk of PICM by 70% in the LBBAP group compared to the RVP group; and (3) two groups showed similar safety profiles both in procedure‐related complications and complications during follow‐up.

Conventional RVP has the potential risk of PICM and hospitalizations for HF in patients who predicted high pacing burden [22, 23]. CSP has been widely conducted to provide more physiological pacing and bring about hemodynamic stability during pacing, which can result in minimizing adverse outcomes [24, 25]. Known risk factors for PICM include older age, presence of HF, lower baseline LVEF, a high percentage of ventricular pacing, and wider paced QRS duration [3, 26, 27, 28]. A recent meta‐analysis reported that upgrading to CSP in patients who developed PICM from RVP led to improvements in left ventricular function and HF symptoms [28]. In this context, recent guidelines recommend CSP for patients with an indication for pacemaker implantation who are expected to require substantial ventricular pacing and have a LVEF greater than 35% [7, 8]. Recent prospective study has demonstrated that LBBAP not only prevents PICM but also improved LVEF in subjects with depressed LV function during median 23 months of follow‐up [29]. This aligns with our findings, further supporting the efficacy of LBBAP as a pacing modality that minimizes the adverse effects of conventional RV pacing and ensures better long‐term ventricular function.

In terms of left ventricular remodeling, LBBAP was associated with a decrease in left ventricular size and NT‐proBNP levels and showed no significant change in LVEF [30]. Sharma et al. compared the risk of composite outcome of all‐cause mortality, hospitalization for HF, or upgrade to biventricular pacing between LBBAP and RVP and reported 54% reduced risk of composite outcome [4]. Tan et al. showed comparable result that CSP was significantly associated with 47% reduced risk of primary outcome and 60% of HF hospitalization [25]. Similarly, a recent previous report based on a large population‐based cohort study using data from the Micra Coverage with Evidence Development study, reported significant benefits of CSP over dual‐chamber RVP, including lower rates of HF hospitalization (HR 0.70; *p* = 0.02) and all‐cause mortality (HR 0.66; *p* < 0.001), particularly with LBBAP [31]. In our study, these findings are consistent that showed 73% reduced risk (absolute risk reduction of 21.2% at 1 year, 18.0% at 2 years, and 12.6% at 3 years) in the composite outcome of PICM, HF hospitalization, and upgrade to CRT compared to the RVP group.

For performing LBBAP, an active helix fixation mechanism and a specially shaped sheath are required to access the septum, where the conduction system is located. SDLs facilitate this technique by eliminating the need for an adapter and allowing for the monitoring of changes in QRS morphology during the screwing of the lead into the septum via the stylet [8, 32]. Their stiffer body and the support provided by the stylet facilitate septal penetration compared to LLLs. However, SDLs have a larger diameter than LLLs due to the space within the lead for the stylet and non‐isodiametric extendable helix design, which can also hinder lead penetration [33]. Additionally, their stiffness may lead to a higher risk of fracture during follow‐up [33, 34]. It has also been reported that the risk of post‐procedural microdislodgement may be higher with SDLs compared to LLLs due to specific characteristics of SDLs [13, 14]. Most procedural experience with CSP, including LBBAP, has been accumulated with LLLs rather than SDLs. Consequently, despite some concerns about the long‐term data for SDLs, the majority of comparative data on LBBAP vs. RVP has been reported from LBBAP cohorts using LLLs. Therefore, only a limited number of studies have reported clinical outcomes of LBBAP using SDLs vs. RVP. In our study, we performed LBBAP by using only SDLs, and showed that performing LBBAP by using SDLs was feasible and safe. Furthermore, in this study, there was no acute and chronic complication of lead fracture during implantation and follow‐up or severe RV lead‐induced tricuspid regurgitation requiring lead removal, and only one case of ventricular lead dislodgement in early after procedure among LBBAP group. This report provides a valuable addition to the existing literature on the comparison of clinical outcomes between LBBAP and RVP.

In actual practice, more than 70% of physicians performed CSP to the patients who have AVB with preserved left ventricular systolic function according to the EHRA survey [10]. In a landscape where CSP dominates pacemaker implantation techniques, it is crucial to appropriately utilize both SDLs and LLLs, each with their distinct advantages and disadvantages. Although existing studies have shown CSP to outperform RVP in terms of clinical outcomes, our study gains significance by demonstrating that LBBAP, when performed exclusively with SDLs, can yield significantly better clinical outcomes compared to RVP. Furthermore, our study highlights the feasibility of conducting LBBAP safely with a low rate of acute complications, reinforcing the versatility and safety of using SDLs in the context of LBBAP.

### 4.1 Study Limitations

First, the observational nature of this study made it impossible to infer causality. Second, this study analyzed relatively small number of patients, so larger multicenter cohort and randomized control study are needed to generalize the findings and identify the ideal pacing modality. Third, the follow‐up duration differs significantly between the two groups, with the LBBAP group having a relatively shorter follow‐up duration. The LBBAP group's median follow‐up duration was 371.0 days (IQR 213.5–557.5), which can be attributed to its later adoption during the study period. Further research is needed into the long‐term follow‐up results of the LBBAP. Fourth, the primary outcome of this study included a soft endpoint, based on echocardiographic findings. This could potentially limit the robustness of the findings. Fifth, the retrospective nature of this study introduces inherent differences between the RVP and LBBAP groups, which may affect the robustness of the findings. Despite adjusting for several key variables, residual confounding cannot be entirely ruled out. Sixth, this study presents the acute success rate; however, it is limited by the lack of long‐term follow‐up pacing data. Lastly, although the LBBAP group using SDLs in our study demonstrated a lower composite event rate compared to the RVP group, our analysis did not include data on how long LBB area capture was maintained during follow‐up in the LBBAP cohort. While recent data in HF patients suggest that LVSP may yield worse outcomes compared to LBBP [18, 35], further studies are needed to assess how changes in the capture area during follow‐up may impact long‐term outcomes in patients with pacing indications and normal EF.

## 5 Conclusions

LBBAP performed with SDL was significantly associated with a lower risk of a composite of PICM, HF hospitalization, and upgrade to CRT than RVP in patients requiring substantial ventricular pacing. In actual clinical practice, LBBAP with SDL has performed safely and effectively compared to RVP.

## Conflicts of Interest

E.K.C.: Research grants or speaking fees from Abbott, Bayer, BMS/Pfizer, Biosense Webster, Chong Kun Dang, Daewoong Pharmaceutical Co., Daiichi‐Sankyo, DeepQure, Dreamtech Co. Ltd., Jeil Pharmaceutical Co. Ltd, Medtronic, Samjinpharm, Samsung Electronics Co. Ltd., Seers Technology, and Skylabs. The others declare no competing interests associated with this manuscript.

## Supporting information

Supporting information.

## Data Availability

Data sharing is not applicable to this article as no new data were created or analyzed in this study.
