# Supplementary material for: Comparison of Clinical Outcomes Between Left Bundle Branch Area Pacing With a Stylet‐Driven Lead and Conventional Right Ventricular Pacing
Source: J Cardiovasc Electrophysiol. 2025 Mar 27;36(6):1253–63. doi: 10.1111/jce.16648 (PMC12160678; doi:10.1111/jce.16648)
Supplement: Supplementary file 1 — Supporting information. [file JCE-36-1253-s001.docx]

**Comparison of clinical outcomes between left bundle branch area pacing with a stylet-driven lead and conventional right ventricular pacing**

Kyung-Yeon Lee, MD^a*^, Jinsun Park, MD^b*^, JungMin Choi, MD^a^, Hyo-Jeong Ahn, MD^a^, Soonil Kwon, MD^c^, Myung-Jin Cha, MD, PhD^b^, Jun Kim, MD, PhD^b^, Gi-Byoung Nam, MD, PhD^b^, Kee-Joon Choi, MD, PhD^b^, Eue-Keun Choi, MD, PhD^a,d^, Seil Oh, MD, PhD^a,d^, Min Soo Cho, MD, PhD^b†^, So‑Ryoung Lee, MD, PhD^a,d†^,

^a^Department of Internal Medicine, Seoul National University Hospital, Seoul, Republic of Korea.

^b^Division of Cardiology, Asan Medical Center, University of Ulsan College of Medicine, Seoul, Republic of Korea.

^c^Division of Cardiology, Department of Internal Medicine, SMG–SNU Boramae Medical Center, Seoul, Republic of Korea.

^d^Department of Internal Medicine, Seoul National University College of Medicine, Seoul, Republic of Korea.

**Supplementary Tables**

**Supplementary Table S1. Clinical outcomes for left bundle branch area pacing and right ventricular pacing groups**

**Supplementary Table S2. Complications for left bundle branch area pacing and right ventricular pacing groups**

**Supplementary Table S3. Sensitivity analysis: restricting follow-up duration for 1-year and 2-year**

**Supplementary Table S4. Sensitivity analysis: in sub-population with atrioventricular block, dual chamber pacing, or not receiving cardiac surgery**

**Supplementary Table S5. Sensitivity analysis excluding patients in the RVP group who underwent RVP after the introduction of LBBAP**

**Supplementary Table S6. Sensitivity analysis: excluding DSP in RVP group**

**Supplementary Figures**

**Supplementary Figure S1. Number of registered patients in each group at 3-month intervals**

|  | **Total (n=738)** | **RVP (n=495)** | **LBBAP (n=243)** | **P-value** |
| --- | --- | --- | --- | --- |
| **Composite outcome I^*^** | 68 (9.2) | 63 (12.7) | 5 (2.1) | <0.001 |
| **Composite outcome II**^†^ | 81 (11.0) | 72 (14.5) | 9 (3.7) | <0.001 |
| **HF hospitalization** | 43 (5.8) | 38 (7.7) | 5 (2.1) | 0.004 |
| **PICM**^‡^ | 46 (6.2) | 42 (8.5) | 4 (1.6) | 0.001 |
| **CRT upgrade** | 7 (0.9) | 7 (1.4) | 0 (0.0) | 0.145 |
| **Cardiovascular death** | 7 (0.9) | 7 (1.4) | 0 (0.0) | 0.145 |
| **All-cause death** | 27 (3.7) | 22 (4.4) | 5 (2.1) | 0.157 |

**Supplementary Table S1. Numbers and percents of patients with clinical outcomes for left bundle branch area pacing and right ventricular pacing groups**

^*^ Composite outcome I included PICM, hospitalization or unplanned hospital visits for HF, and device upgrade to CRT.

^†^ Composite outcome II included PICM, hospitalization or unplanned hospital visits for HF, device upgrade to CRT and all-cause death.

^‡^ PICM was defined as a decline in LVEF from normal to EF<50% or a decrease of more than 10% (as an absolute value) from baseline.

Abbreviations: CRT, cardiac resynchronization therapy; HF, heart failure; LBBAP, left bundle branch area pacing; PICM, pacing-induced cardiomyopathy; RV, right ventricular; RVP, right ventricular pacing.

**Supplementary Table S2. Complications for left bundle branch area pacing and right ventricular pacing groups**

|  | **Total (n=738)** | **RVP (n=495)** | **LBBAP (n=243)** | **P-value** |
| --- | --- | --- | --- | --- |
| **Acute** |  |  |  |  |
| Ventricular lead related complication ^*^ | 0 | 0 | 0 | - |
| Pneumothorax | 3 (0.4) | 1 (0.2) | 2 (0.8) | 0.528 |
| Pericardial effusion ^†^ | 4 (0.5) | 4 (0.8) | 0 (0) | 0.383 |
| Pocket related complication ^‡^ | 2 (0.3) | 2 (0.4) | 0 (0.0) | 0.811 |
| **Chronic** |  |  |  |  |
| Need for ventricular lead revision | 18 (2.4) | 15 (3.0) | 3 (1.2) | 0.218 |

^*^ Ventricular lead related complication was defined as lead fracture during implantation or severe right ventricular lead-induced tricuspid regurgitation requiring lead removal.

^†^ All cases required pericardiocentesis, with one case involving tamponade.
^‡^ Pocket related complication was defined as an infection of the pocket or lead, or the occurrence of a pocket hematoma that required additional intervention.

Abbreviations: LBBAP, left bundle branch area pacing; RVP, right ventricular pacing.

**Supplementary Table S3. Sensitivity analysis: restricting follow-up duration for 1-year and 2-year**

|  | **Event / Total N** | **Incidence Rate (100PY)** | **aHR (95% CI)** | **P-value** |
| --- | --- | --- | --- | --- |
| **Restricting follow-up to 1 year** |  |  |  |  |
| **Composite outcome I^*^** |  |  |  |  |
| **RVP** | 11/119 | 18.9 | 1 (Reference) | - |
| **LBBAP** | 1/119 | 1.47 | 0.04 (0.00 – 0.37) | 0.004 |
| **Composite outcome II**^†^ |  |  |  |  |
| **RVP** | 14/119 | 24.6 | 1 (Reference) | - |
| **LBBAP** | 5/119 | 7.45 | 0.29 (0.09 – 0.96) | 0.043 |
| **Restricting follow-up to 2 years** |  |  |  |  |
| **Composite outcome I^*^** |  |  |  |  |
| **RVP** | 25/229 | 11.8 | 1 (Reference) | - |
| **LBBAP** | 5/233 | 2.24 | 0.20 (0.07 – 0.53) | 0.001 |
| **Composite outcome II**^†^ |  |  |  |  |
| **RVP** | 31/229 | 14.6 | 1 (Reference) | - |
| **LBBAP** | 9/233 | 4.03 | 0.30 (0.13 – 0.067) | 0.003 |

Adjusted by age, sex, hypertension, diabetes mellitus, heart failure, valvular heart disease, atrial fibrillation, diuretics, baseline QRS duration, and underlying bundle branch block.

**^*^** Composite outcome I included PICM, hospitalization or unplanned hospital visits for HF, and device upgrade to CRT.

^†^ Composite outcome II included PICM, hospitalization or unplanned hospital visits for HF, device upgrade to CRT and all-cause death.

Abbreviations: CI, confidence interval; HR, hazard ratio; PY, person-years.

**Supplementary Table S4. Sensitivity analysis: in sub-population with atrioventricular block, dual chamber pacing, or not receiving cardiac surgery**

|  |  | **Event / N** | **IR (PY)** | **aHR (95% CI)** | **P-value** |
| --- | --- | --- | --- | --- | --- |
| **AVB** | **Composite outcome I^*^** |  |  |  |  |
|  | **RVP** | 33/343 | 0.05 | 1 (Reference) | - |
|  | **LBBAP** | 3/214 | 0.01 | 0.21 (0.06 - 0.71) | 0.012 |
|  | **Composite outcome II**^†^ |  |  |  |  |
|  | **RVP** | 38/343 | 0.06 | 1 (Reference) | - |
|  | **LBBAP** | 7/214 | 0.03 | 0.43 (0.19 – 0.98) | 0.043 |
|  | **PICM** |  |  |  |  |
|  | **RVP** | 25/343 | 0.04 | 1 (Reference) | - |
|  | **LBBAP** | 2/214 | 0.01 | 0.19 (0.04 - 0.80) | 0.024 |
| **Dual chamber pacing** | **Composite outcome I^*^** |  |  |  |  |
|  | **RVP** | 40/367 | 0.06 | 1 (Reference) | - |
|  | **LBBAP** | 3/222 | 0.01 | 0.17 (0.05 - 0.55) | 0.003 |
|  | **Composite outcome II**^†^ |  |  |  |  |
|  | **RVP** | 43/367 | 0.07 | 1 (Reference) | - |
|  | **LBBAP** | 7/222 | 0.03 | 0.36 (0.16 – 0.83) | 0.016 |
|  | **PICM** |  |  |  |  |
|  | **RVP** | 28/367 | 0.04 | 1 (Reference) | - |
|  | **LBBAP** | 2/222 | 0.01 | 0.15 (0.04 - 0.65) | 0.011 |
| **No cardiac surgery** | **Composite outcome I^*^** |  |  |  |  |
|  | **RVP** | 46/392 | 0.06 | 1 (Reference) | - |
|  | **LBBAP** | 4/195 | 0.02 | 0.22 (0.08 - 0.61) | 0.004 |
|  | **Composite outcome II**^†^ |  |  |  |  |
|  | **RVP** | 52/392 | 0.07 | 1 (Reference) | - |
|  | **LBBAP** | 7/195 | 0.04 | 0.35 (0.16 – 0.79) | 0.011 |
|  | **PICM** |  |  |  |  |
|  | **RVP** | 31/392 | 0.04 | 1 (Reference) | - |
|  | **LBBAP** | 3/195 | 0.02 | 0.24 (0.07 - 0.79) | 0.019 |

Adjusted by age, sex, hypertension, diabetes mellitus, heart failure, valvular heart disease, atrial fibrillation, diuretics, baseline QRS duration, and underlying bundle branch block.

**^*^**Composite outcome I included PICM, hospitalization or unplanned hospital visits for HF, and device upgrade to CRT.

^†^Composite outcome II included PICM, hospitalization or unplanned hospital visits for HF, device upgrade to CRT and all-cause death.

Abbreviations: AVB, atrioventricular block; CI, confidence interval; HR, hazard ratio; IR, incidence rate; LBBAP, left bundle branch area pacing; PICM, pacing-induced cardiomyopathy; PY, person-year; RVP, right ventricular pacing.

**Supplementary Table S5. Sensitivity analysis excluding patients in the RVP group who underwent RVP after the introduction of LBBAP**

|  | **Event / N** | **IR (PY)** | **aHR (95% CI)** | **P-value** |
| --- | --- | --- | --- | --- |
| **Composite outcome I^*^** |  |  |  |  |
| **RVP** | 31/196 | 5.96 | 1 (Reference) | - |
| **LBBAP** | 5/243 | 2.06 | 0.30 (0.10 - 0.90) | 0.031 |
| **Composite outcome II**^†^ |  |  |  |  |
| **RVP** | 34/196 | 6.54 | 1 (Reference) | - |
| **LBBAP** | 9/243 | 3.70 | 0.45 (0.18 - 1.11) | 0.083 |
| **PICM** |  |  |  |  |
| **RVP** | 22/196 | 4.17 | 1 (Reference) | - |
| **LBBAP** | 4/243 | 1.63 | 0.37 (0.11 - 1.25) | 0.111 |

Adjusted by age, sex, hypertension, diabetes mellitus, heart failure, valvular heart disease, atrial fibrillation, diuretics, baseline QRS duration, and underlying bundle branch block

**^*^**Composite outcome I included PICM, hospitalization or unplanned hospital visits for HF, and device upgrade to CRT.

^†^Composite outcome II included PICM, hospitalization or unplanned hospital visits for HF, device upgrade to CRT and all-cause death.

Abbreviations: CI, confidence interval; HR, hazard ratio; IR, incidence rate; LBBAP, left bundle branch area pacing; PICM, pacing-induced cardiomyopathy; PY, person-year; RVP, right ventricular pacing.

**Supplementary Table S6. Sensitivity analysis excluding DSP in RVP group**

|  | **Event / N** | **IR (PY)** | **aHR (95% CI)** | **P-value** |
| --- | --- | --- | --- | --- |
| **Composite outcome I^*^** |  |  |  |  |
| **RVP** | 61/487 | 6.68 | 1 (Reference) | - |
| **LBBAP** | 5/243 | 2.06 | 0.25 (0.09 - 0.64) | 0.004 |
| **Composite outcome II**^†^ |  |  |  |  |
| **RVP** | 70/487 | 7.67 | 1 (Reference) | - |
| **LBBAP** | 9/243 | 3.70 | 0.41 (0.20 – 0.85) | 0.016 |
| **PICM** |  |  |  |  |
| **RVP** | 40/487 | 4.31 | 1 (Reference) | - |
| **LBBAP** | 4/243 | 1.65 | 0.30 (0.10 - 0.88) | 0.029 |

Adjusted by age, sex, hypertension, diabetes mellitus, heart failure, valvular heart disease, atrial fibrillation, diuretics, baseline QRS duration, and underlying bundle branch block.

**^*^**Composite outcome I included PICM, hospitalization or unplanned hospital visits for HF, and device upgrade to CRT.

^†^Composite outcome II included PICM, hospitalization or unplanned hospital visits for HF, device upgrade to CRT and all-cause death.

Abbreviations: CI, confidence interval; HR, hazard ratio; IR, incidence rate; LBBAP, left bundle branch area pacing; PICM, pacing-induced cardiomyopathy; PY, person-year; RVP, right ventricular pacing.

**Supplementary Figure S1. Number of registered patients in each group at 3-month intervals**  **
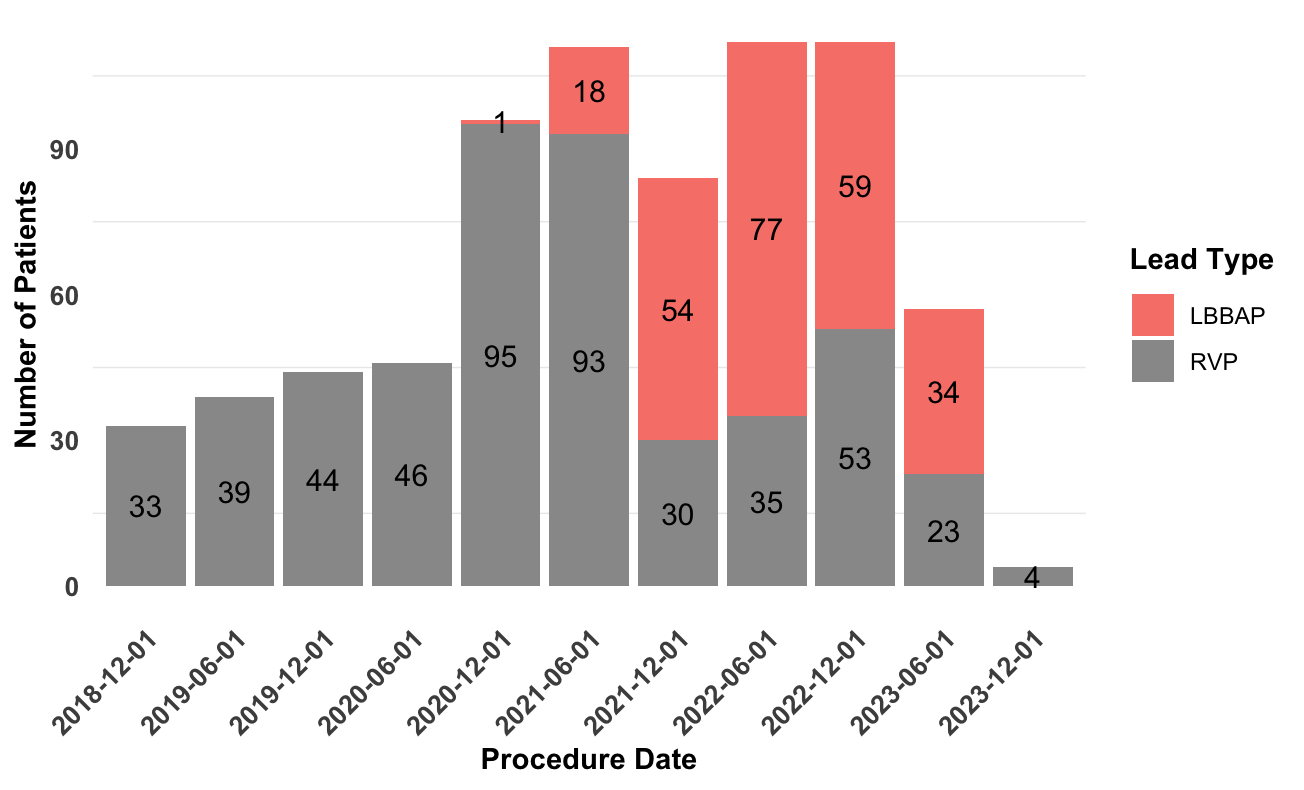
**

Abbreviations: LBBAP, left bundle branch area pacing; RVP, right ventricular pacing.
